# Editorial: Development of novel reagents to reverse drug resistance in bacterial pathogens

**DOI:** 10.3389/fcimb.2022.1047108

**Published:** 2022-10-11

**Authors:** Yasheng Li, Lisheng Liao, Shicheng Chen

**Affiliations:** ^1^ Department of Infectious Diseases & Anhui Center for Surveillance of Bacterial Resistance, The First Affiliated Hospital of Anhui Medical University, Hefei, China; ^2^ Guangdong Laboratory of Microbial Signals and Disease Control, Integrative Microbiology Research Centre, South China Agricultural University, Guangzhou, China; ^3^ Clinical and Diagnostic Sciences, School of Health Sciences, Oakland University, Rochester, MI, United States

**Keywords:** resistance, pathogens, anti-MDR, inhibitors, mechanisms

Antimicrobial resistance is one of the top ten threats to public health according to the World Health Organization. The multiple drug resistance mechanisms have been well documented in bacterial pathogens including the production of hydrolytic enzymes passivating enzymes and modifying enzymes, target changes of antibiotic action, decreased permeability of bacterial membranes, including the formation of bacterial biofilm and loss of channel proteins, and overexpression of efflux systems. In this Research Topic, four works (two original research articles and two reviews) explored novel bacterial resistance traits. Discoveries included tubo-ovarian abscess (TOA), bacterial infection and antibiotics in Parkinson’s disease, multidrug-resistant *Pseudomonas aeruginosa* in non-cystic fibrosis bronchiectasis (NCFB), the use of auranofin (AUR) and phenethyl isothiocyanate (PEITC) against *Staphylococcus aureus* Skin Infection.

Tang et al. reviewed the resistance mechanisms of the main pathogenic bacteria causing TOA, including the production of hydrolytic or modifying enzymes, the reduction of cell membrane permeability, efflux pump mediation, ribosome protection, and target site mutations. They suggested an in-depth study of the specific drug resistance mechanism involved in female genital tract infections is particularly warranted. Furthermore, they proposed new treatment approaches with narrow-spectrum drugs to target the specific causative agents, which will avoid super infections.

Sheng et al. reviewed the relationships between common bacterial infections (*H. pylori*, *M. tuberculosis*, *P. gingivalis*, *C. difficile* and *C. pneumoniae*) in Parkinson’s disease (PD) patients and their possible action mechanisms. Neuroinflammation factors, LRRK2 pathway and toxic protein aggregations were highlighted in this review.

Ding et al., through a comprehensive analysis of 97 P*. aeruginosa* isolates from clinical NCFB patients, found that carbapenem use during treatment was directly associated with isolates being MDR strains. MDR isolates demonstrated an absence of a quorum sensing (QS) system which can be detected in non-MDR isolates. Further, they showed that exogenous addition of the QS signaling molecule 3OC12-HSL could not complement this phenotype back. Interestingly, the efflux pump inhibitor PAβN not only restores the QS phenotype in MDR isolates, but also delays the early emergence of lasR mutants in evolutionary experiments. They proposed that MDR *P. aeruginosa* possibly mediated the lasR mutation through up-regulation of efflux pumps. Anti-QS combined with an efflux pump inhibitor strategy may be a potential strategy to treat MDR *P. aeruginosa* infection in NCFB patients.

The development of novel therapies by using old drugs may alleviate the problems in antibiotic resistance in *S. aureus*. Chen et al. showed that continuous monotherapy by using auranofin (AUR) leads to low susceptibility or even develops drug resistance in *S. aureus*. They proposed a novel treatment regime by applying AUR and phenethyl isothiocyanate (PEITC) to manage *S. aureus* skin infections. They reported there was a synergistic effect by checkerboard test and time-kill kinetic analysis due to increased reactive oxygen species (ROS) generation, disruption of bacterial cellularity, and inhibition of biofilm formation. It is worth mentioning that they found that this drug combination effectively restored susceptibility to AUR by regulating thioredoxin reductase (TrxR) and protected mice from subcutaneous abscesses by eliminating *S. aureus* including methicillin-resistant *S. aureus* (MRSA).

In conclusion, understanding the mechanisms of novel drug-resistant resistance will allow to develop novel drugs such as quorum sensing inhibitors, effector protein inhibitors and biofilm development inhibitors. This Research Topic provides some guidance for summarizing the latest progress of studying bacterial resistance mechanisms and developing novel management strategies to avoid MDR.

## Author contributions

YL, SC and LL drafted the manuscript. These authors reviewed and approved the final manuscript.

## Funding

This work was supported by the National Natural Science Foundation of China (Grant No. 82204202 and Grant No. 31900076).

## Acknowledgments

Editors thank the authors for their contributions, and all the reviewers for their effort and expertise that significantly contributed to the quality of this Research Topic.

## Conflict of interest

The authors declare that the research was conducted in the absence of any commercial or financial relationships that could be construed as a potential conflict of interest.

## Publisher’s note

All claims expressed in this article are solely those of the authors and do not necessarily represent those of their affiliated organizations, or those of the publisher, the editors and the reviewers. Any product that may be evaluated in this article, or claim that may be made by its manufacturer, is not guaranteed or endorsed by the publisher.

